# The association between serum adipocyte fatty acid–binding protein and 3-month disability outcome after aneurysmal subarachnoid hemorrhage

**DOI:** 10.1186/s12974-020-01743-2

**Published:** 2020-02-19

**Authors:** Yong-Gang Luo, Bing Han, Tong-Wen Sun, Xianzhi Liu, Jun Liu, Jun Zhang

**Affiliations:** 1 Department of Intensive Care Unit, The First Affiliated Hospital of Zhengzhou University, Zhengzhou, 450000 Henan China; 2 Department of Neurosurgery, The First Affiliated Hospital of Zhengzhou University, Zhengzhou, 450000 Henan China; 3 Department of Pharmacy, The First Affiliated Hospital of Zhengzhou University, No. 1, Jianshe East Road, Zhengzhou, 450000 Henan China

**Keywords:** Adipocyte fatty acid–binding protein, Aneurysmal subarachnoid hemorrhage, Prognostic, Functional outcome, Mortality, Chinese

## Abstract

**Background:**

Adipocyte fatty acid–binding protein (FABP4) is an adipokine that plays an important role in development of cardiovascular and metabolic diseases. The aim of this study was to assess the 3-month prognostic value of serum levels of FABP4 in Chinese patients with aneurysmal subarachnoid hemorrhage (aSAH) on hospital admission.

**Methods:**

This was a prospective observational study from a stroke treatment center in Zhengzhou, China. From October 2016 to May 2018, patients with aSAH who were hospitalized within 24 h were included. In addition, 202 age- and gender-matched healthy volunteers were assigned to the healthy control group. At admission, serum levels of FABP4 were measured, and patients’ characteristics, Hunt–Hess grade, and modified Fisher grade evaluated. At 3-month follow-up, functional outcome (Glasgow Outcome Scale score; dichotomized as poor [score 1–3] or good [score 4–5]) and all-cause mortality were recorded. Univariate and multivariate logistic regression models were used to investigate the association of FABP4 with the two endpoints.

**Results:**

A total of 418 patients with aSAH were included in this study. The median age was 58 years (interquartile range, 49–66 years), and 57.9% were women. FABP4 serum levels were related to Hunt–Hess score (*r*[Spearman] = 0.381; *P* < 0.001). Patients with a poor outcome and non-survivors had significantly increased serum FABP4 levels on admission (*P* < 0.001 for all). In multivariate logistic regression analysis, FABP4 was an independent predictor of poor outcome and mortality, with increased risks of 7% (odds ratios 1.07, 95% confidence interval [CI] 1.02–1.13; *P* = 0.001) and 5% (odds ratio 1.05, 95% CI, 1.01–1.12; *P* = 0.003), respectively. Receiver operating characteristics to predict functional outcome and mortality were significantly different between conventional risk factors (difference area under the curve 0.024, 95% CI 0.018–0.032) and FABP4 plus conventional risk factors (area under the curve 0.015, 95%CI 0.011–0.020). After FABP4 was added to the existing risk factors, mortality was better reclassified and was associated with the net reclassification improvement statistic (*P* = 0.009), while poor outcome was better reclassified and associated with both the integrated discrimination improvement and net reclassification improvement statistics (*P* < 0.05 for all).

**Conclusions:**

Elevated serum FABP4 levels were related to poor outcome and mortality in a cohort of patients with aSAH.

## Background

Subarachnoid hemorrhage (SAH), a devastating form of stroke, continues to be a serious and significant health problem in China and worldwide [1]. The most common type of SAH is aneurysmal SAH (aSAH), which accounts for > 80% of cases [2]. An increasing trend of SAH incidence over time was noted in Hong Kong, while the 1-year mortality rates decreased from 43 to 19% (2002–2010) [3]. Lantigua et al. [4] also reported an in-hospital mortality of SAH of 18% (216/1200 cases), with the most common primary causes of death or neurological devastation leading to withdrawal of support including primary hemorrhage (55%), aneurysm rebleeding (17%), and medical complications (15%). In addition, many SAH patients (> 50%) who had early good neurological recovery experienced reintegration difficulties after 20 years [5]. However, the causes and mechanisms of brain injury and death after SAH in the modern era of neurocritical care remain poorly understood [4]. Biomarkers can be used to predict patient risk and act as an early warning indictor of delayed ischemic injury [5].

Fatty acid–binding protein 4 (FABP4), also known as adipocyte FABP (A-FABP) or aP2, is mainly expressed in adipocytes and macrophages and plays an important role in the development of insulin resistance and atherosclerosis following metaflammation (low-grade and chronic inflammation) [6]. Elevated serum levels of FABP4 are also associated with obesity [7], diabetes mellitus [8], hypertension [9], and cardiovascular events [10]. Rodríguez-Calvo reported that FABP4 was a useful biomarker in atherosclerosis and coronary artery disease and was directly related to cardiac alterations [11]. Further, Tso et al. [12] showed that serum FABP4 was independently related to stroke (odds ratio [OR] 2.10, 95% confidence interval [CI] 1.50–2.94; *P* < 0.001), and a high serum level was associated with increased risk of 3-month mortality in ischemic stroke subjects (OR 2.65, 95% CI 1.18–5.96; *P* = 0.018). High levels of FABP4 were also significantly associated with stroke risk and severity, independent from other risk factors [13], while elevated serum FABP4 levels were associated with poor prognosis in ischemic stroke patients with type 2 diabetes [14].

However, the clinical significance of FABP4 in patients with SAH remains unclear. Thus, we examined the hypothesis that high levels of serum FABP4 are related to poor prognosis in patients with SAH. The aim of this study was to assess the 3-month prognostic value of serum levels of FABP4 in Chinese patients with aneurysmal SAH (aSAH) on hospital admission.

## Patients and methods

### Participants and study design

This was a prospective observational study from a stroke treatment center in Zhengzhou, China. All patients ≥ 18 years of age with a newly diagnosed aSAH admitted to our hospital from October 2015 to May 2018 were offered enrollment into this study. The exclusion criteria included (1) bleeding occurring more than 24 h before admission, (2) SAH from other secondary causes (antecedent head trauma, ischemic or hemorrhagic stroke, and vascular malformation), and (3) systemic diseases (chronic neurological disease, tumors, liver and/or renal insufficiency, and chronic lung disease). All included patients underwent computed tomography (CT) angiography or conventional cerebral angiography and received treatment according to clinical treatment guidelines.

At the same time, 202 age- and gender-matched healthy volunteers were assigned to the healthy control group. The median age of the control cases was 58 years (interquartile range [IQR] 49–66), and 58.4% were women.

### Clinical definitions

Medical and surgical management was performed as appropriate. Following our institutional protocol, prophylactic antiepileptic medication was administered for 1 week after SAH. For each patient, data on demographics (age, sex, and race), body mass index (BMI), and time from disease onset to admission were collected. Basic comorbidities (hypertension, diabetes, and cardiovascular diseases), smoking, drinking, and complications during treatment were identified. Aneurysm size, location, surgical time, and the surgical technique (surgical clip vs. endovascular coil) were collected according to hospital records. The Acute Physiology and Chronic Health Evaluation II score and the presence of mechanical ventilation were recorded.

The severity of admission neurologic grade was analyzed by the Hunt–Hess (H-H) score (range from 0 to 5) [15]. The H-H scale was used as the instrument for grading neurologic impairment (severe grade was defined as 4–5, while non-severe grade was defined as 1–3). CT scans were assessed by neurointensivists for the extent of initial bleeding using the modified Fisher scale [16] and intraventricular hemorrhage score [17]. Transcranial Doppler (TCD) ultrasound was performed every day until 21 days after hospitalization for all patients studied. Cerebral vasospasm was confirmed by TCD according to two criteria: mean velocities in the middle cerebral arteries > 120 cm/s and Lindegaard ratios > 3 [15, 18]. Intra-arterial nimodipine was used if cerebral vasospasm was confirmed during angiography. CT scans were used according to patient’s clinical presentation or at least once during hospitalization to exclude post-hemorrhagic hydrocephalus [19]. Delayed cerebral ischemia (DCI) was diagnosed by a neurologist (HB) according to the flowing criteria: (1) a new focal neurological deficit and/or a decrease in the level of consciousness, lasting for > 1 h, and (2) a new infarct (confirmed by CT) that was not visible on admission or on the immediate postoperative scan. Hydrocephalus was classified as present or absent based on the bicaudate index according to the upper limit of normal for the age decile [20].

### Outcome assessment

All included patients received a follow-up at 3-months after admission. Functional outcome was assessed by the Glasgow Outcome Scale (GOS) score (ranging from 1 to 5 with ascending grade of recovery) [21]. The primary end point was good functional outcome (defined as a GOS score of 4–5 points) [19]. Secondary end point was all-cause death within follow-up. Outcome evaluation was completed by one researcher (ST) and two trained nurses (not the authors) with a structured telephone interview with the patient or with the relatives. This assessment process was blinded for FABP4 levels.

### Blood sample testing

Fasting blood samples were obtained from the cubital vein on the first morning after admission and within 48 h of SAH onset. Serum samples were separated and stored at − 70 °C. Serum levels of glucose and C-reactive protein (CRP) were measured by standard detection methods. Serum concentrations of FABP4 were tested by a commercially available enzyme-linked immunosorbent assay (R&D Systems, Minneapolis, MN, USA). The inter-assay and intra-assay coefficients of variation were 5.0–8.5% and 3.2–5.1% at a concentration between 10 and 100 ng/ml, respectively.

### Statistical analysis

A normal distribution test was performed with the Shapiro–Wilk test. Categorical variables are presented as number and percentages, while continuous variables are presented as medians and IQRs. Two-group comparisons were performed using the Mann–Whitney *U* test (continuous variables) or the *χ*^2^ test (categorical variables). Spearman’s rank correlation was analyzed by bivariate correlations. The relationship between FABP4 and severe SAH (defined as H-H grades 4 and 5) was assessed by univariate and multivariate (adjusted for age, sex, BMI, ethnicity, time from SAH to admission, hypertension, cardiovascular comorbidities, diabetes, smoking, drinking, aneurysm size and location, serum CRP, and glucose) logistic regression analysis. The relationship between biomarkers and the two endpoints (poor outcome [defined as a GOS score of 1–3 points] and mortality) was also investigated using regression analysis. Crude and adjusted results are presented as OR and 95% CI, respectively. The relationship between FABP4 and endpoints was assessed by the FABP4 quartiles (the lowest quartile [Q1] as the reference). FABP4 levels were also used as a categorical variable (elevated [defined as ≥ 24.9 ng/ml; 3rd quartile] vs. normal) in the regression analysis.

The accuracy of FABP4 in predicting poor outcome and mortality was evaluated by receiver operating characteristic curves, and the data are presented as the area under the curve (AUC). The cutoff of FABP4 value was also confirmed using this method. The effect of adding FABP4 into the conventional risk factors for predicting prognosis was further assessed by the integrated discrimination improvement index and the net reclassification improvement (NRI) index [22]. Finally, the cumulative mortality was determined using Kaplan–Meier survival curves according to FABP4 quartiles and compared with the log-rank test.

All statistical analyses were performed using statistical software (SPSS Statistics v23.0; IBM Corp., Armonk, NY, USA; and the ROCR package v1.0-2, http://cran.r-project.org/). The level of significance was set at 0.05 (two-sided).

### Ethical review

The research protocol for this study was reviewed and approved by the Human Research Ethics Committee (HREC) of the First Affiliated Hospital of Zhengzhou University. Written informed consents were received from all patients according to the guidance of Declaration of Helsinki when they participated in this study.

## Results

### Basic information

**Fig. 1:**
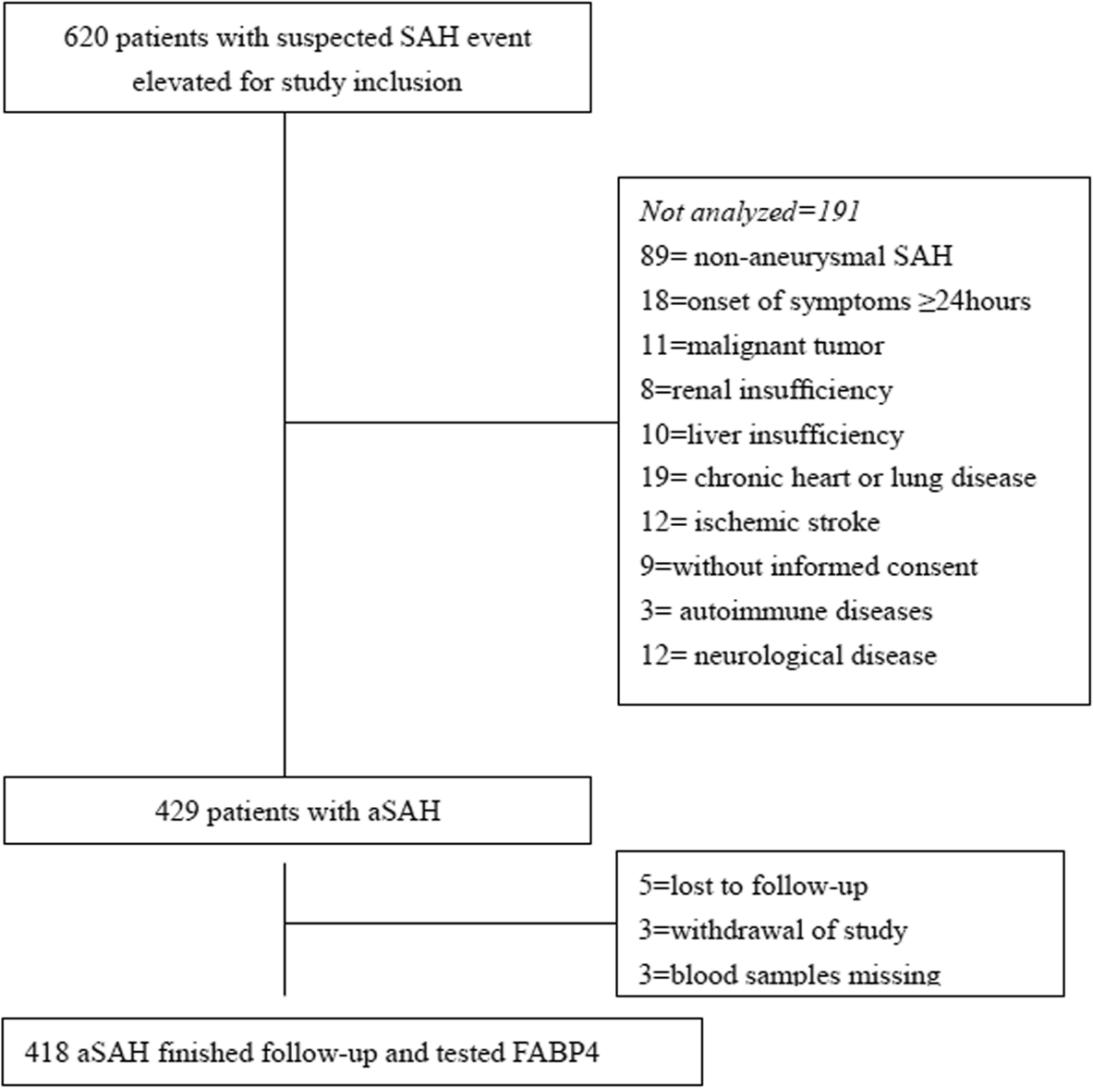
Study profile/flow sheet of the study

**Fig. 2:**
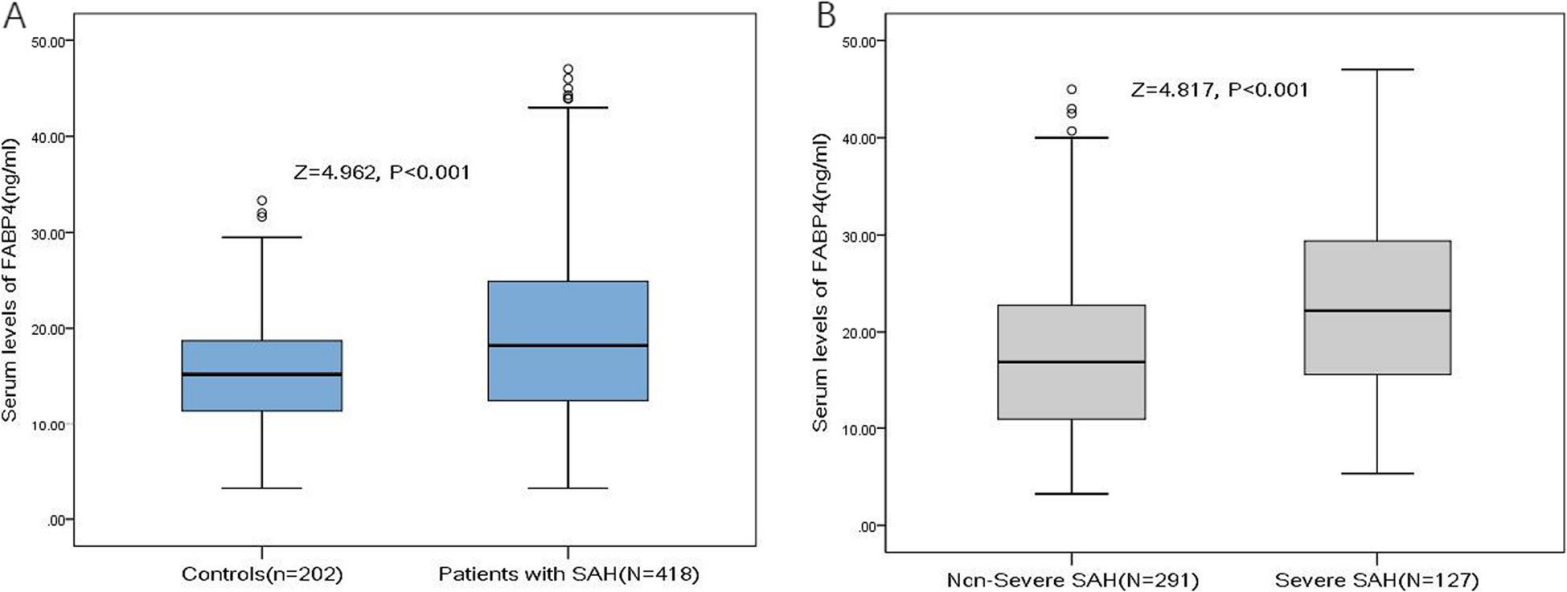
Serum levels of FABP4 in different groups. **a** Serum levels of FABP4 in SAH patients and controls. **b** Serum levels of FABP4 in patients with severe SAH and non-severe SAH. Severe SAH was assessed using the Hunt–Hess score and defined as H-H score 4 or 5. SAH = subarachnoid hemorrhage; FABP4 = fatty acid–binding protein 4

Four hundred and eighteen patients with aSAH were included in the final analysis (Fig. 1). The median age of the patients was 58 years (IQR 49–66), and 57.9% were women. The median serum level of FABP4 in those patients was 18.2 ng/ml (IQR 12.5–24.9), which was higher than that in controls (15.2 ng/ml [IQR 11.4–18.7]; Fig. 2a). The baseline characteristics of the patients are shown in Table 1.

**Table 1:** The included patient characteristics

Variable	Patients with aSAH
*N*	418
Age, years, median (IQR)	58 (49–66)
Sex—women, *n* (%)	242 (57.9)
Ethnicity—Han, *n* (%)	369 (88.3)
BMI, kg/m^2^, median (IQR)	26.1 (24.2–27.3)
The time from SAH to admission, hours, median (IQR)	16.5 (11.5–23.0)
Hypertension, *n* (%)	184 (44.0)
Cardiovascular comorbidities, *n* (%)	59 (14.1)
Diabetes, *n* (%)	73 (17.5)
A history of nicotine abuse, *n* (%)	64 (15.3)
A history of ethanol abuse, *n* (%)	55 (13.2)
Surgical clip, *n* (%)	216 (51.7)
Coiling, *n* (%)	189 (45.2)
Location, *n* (%)	
Anterior cerebral artery and its branches	244 (58.4)
The medial cerebral artery	85 (20.3)
The posterior circulation	89 (21.3)
Complications in the study, *n* (%)	126 (30.1)
Surgical time, min, median (IQR)	145 (85–177)
Mechanical ventilation, *n* (%)	97 (23.2)
Hunt–Hess grade, *n* (%)	
1: Mild headache	83 (19.9)
2: Severe headache	109 (26.0)
3: Lethargy, mild focal deficits	99 (23.7)
4: Stupor	69 (16.5)
5: Coma	58 (13.9)
Modified Fisher grade, *n* (%)	
1: No thick cisternal blood, −IVH	91 (21.8)
2: No thick cisternal blood, +IVH	110 (26.3)
3: Thick cisternal blood, −IVH	118 (28.2)
4: Thick cisternal blood, +IVH	99 (23.7)
Aneurysm size, mm, median (IQR)	6.5 (5.0–9.3)
Aneurysm size > 10 mm, *n* (%)	83 (19.9)
ICH, *n* (%)	105 (25.1)
IVH sum score^a^, median (IQR)	3 (0–6)
APACHE-II score^b^, median (IQR)	16 (10–22)
Hydrocephalus, *n* (%)	103 (24.6)
TCD cerebral vasospasm, *n* (%)	92 (22.0)
DCI, *n* (%)	89 (21.3)
Laboratory findings at admission	
Glucose, mmol/l	6.46 (5.76–6.85)
CRP, mg/l	6.88 (5.15–10.32)
FABP4, ng/ml	18.2 (12.5–24.9)

Values are expressed as numbers (% of total) or median (IQR). Laboratory values reflect admission value

*APACHE-II* 5 Acute Physiology and Chronic Health Evaluation II, *IQR* interquartile range, *IVH* intraventricular hemorrhage, *SAH* subarachnoid hemorrhage, *DCI* delayed cerebral ischemia, *ICH* intracerebral hemorrhage, *CRP* C-reactive protein, *TCD* transcranial Doppler, *aSAH* aneurysmal subarachnoid hemorrhage

^a^Range: 0, no IVH; 12, all ventricles completely filled with IVH

^b^Range: 0–71. Higher scores indicate more severe disease

### Serum levels of FABP4 and SAH severity

**Fig. 3:**
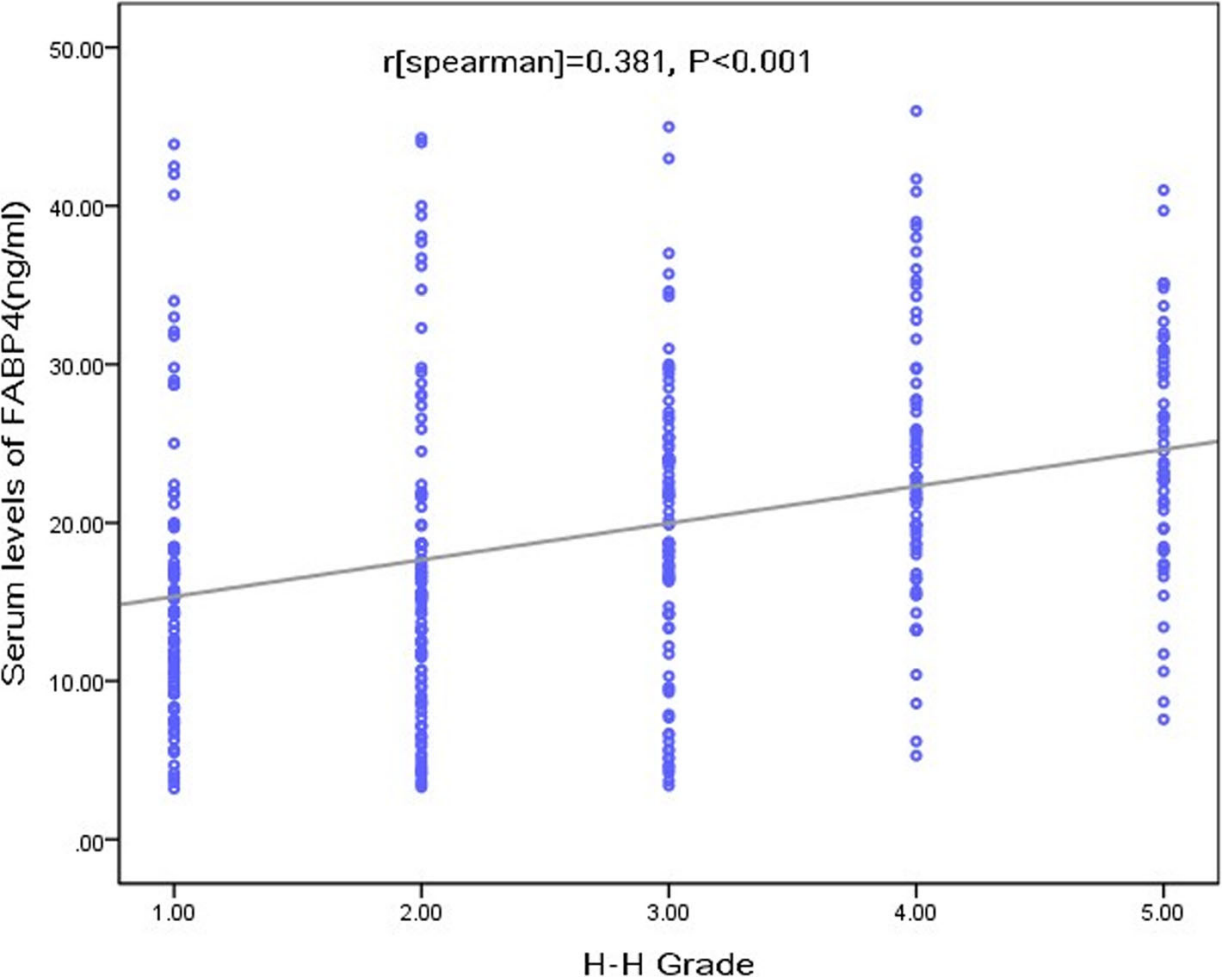
The relationship between serum levels of FABP4 and severity of SAH. Severity of admission neurologic grade was assessed using the Hunt–Hess score. SAH = subarachnoid hemorrhage; FABP4 = fatty acid–binding protein 4

There was a positive correlation of H-H score with FABP4 serum level (*r*[Spearman] = 0.381; *P* < 0.001; Fig. 3). As a categorical variable, patients with severe SAH (defined as an H–H score of 4–5) had higher serum FABP 4 levels compared with those with non-severe SAH (22.2 ng/ml [IQR 15.6-29.5] vs. 16.9 ng/ml [IQR 10.9-22.9], respectively; Fig. 2b). In the univariate model, FABP4 was associated with an increased risk of severe SAH (OR 1.06, 95% CI 1.03–1.08; *P* < 0.001). In multivariable regression analysis, FABP4 was still associated with an increased risk of severe SAH (OR 1.03, 95% CI 1.01–1.06; *P* = 0.008) after adjusting for age, sex, BMI, ethnicity, the time from SAH to admission, hypertension, cardiovascular comorbidities, diabetes, nicotine and ethanol abuse, aneurysm size and location, and serum levels of CRP and glucose. Further, there was a positive correlation of the modified Fisher grade with FABP4 (*r* = 0.402; *P* < 0.001).

aSAH patients were also dichotomized as high blood clot burden (modified Fisher grade 3–4, *n* = 217) and low blood clot burden (modified Fisher grade 1–2, *n* = 201). Patients with a higher initial blood clot more frequently presented with more severe H-H grades than in patients with a low blood clot burden (36.9% vs. 23.4%, respectively; *P* = 0.003). Further, there was a positive correlation of H-H score with the modified Fisher grade (*r* = 0.192; *P* = 0.008). FABP4 was also an independent predictor of severe SAH (OR 1.03, 95% CI 1.01–1.07; *P* = 0.009) after adjustment for both the modified Fisher grade score and the above factors.

The serum levels of FABP4 were associated with inflammatory markers, and there was a positive correlation of FABP4 with CRP (*r* = 0.305; *P* < 0.001). Patients with fever had higher levels of FABP4 than those without fever (*P* = 0.009). Similarly, patients with infectious hospital complications had higher levels of FABP4 compared with those without infectious hospital complications (*P* = 0.003).

### Association of serum levels of FABP4 with poor outcomes

At follow-up, 115 patients were defined as poor outcome (GOS score of 4 or 5). Patients who experienced poor outcomes were older, more likely suffered from hypertension, cardiovascular comorbidities, and nicotine abuse, and had a more severe H-H grade, larger aneurysm size, and higher serum levels of glucose and CRP compared with those who presented with good outcomes (Table 2). Further, patients with poor outcomes were more likely to suffer from hydrocephalus, TCD cerebral vasospasm, and DCI during treatment.

**Table 2:** Univariate and multivariate logistic regression analysis of predictors for poor outcomes

Predictors	Univariate analysis		Multivariate analysis^a^	
	OR (95% CI)	*P*	OR (95% CI)	*P*
Age (per unit increase)	1.18 (1.10–1.25)	0.001	1.09 (1.03–1.17)	0.009
Sex (male vs. female)	1.21 (0.90–1.55)	0.43	–	
Han vs. others	0.93 (0.82–1.33)	0.28	–	
BMI (per unit increase)	1.06 (0.93–2.03)	0.83	–	
The time from SAH to admission (per unit increase)	1.14 (0.88–2.02)	0.17	–	
Hypertension	1.91 (1.31–3.06)	0.013	1.55 (1.04–2.43)	0.063
Diabetes	1.30 (0.89–2.04)	0.19	–	
Cardiovascular comorbidities	1.69 (1.21–2.55)	0.032	1.30 (0.90–1.99)	0.12
A history of nicotine abuse	1.17 (1.03–1.36)	0.024	1.05 (0.98–1.31)	0.22
A history of ethanol abuse	0.74 (0.50–1.22)	0.48	–	
Surgical clip	0.69 (0.61–0.78)	0.002	0.73 (0.60–0.86)	0.010
Coiling	0.84 (0.75–0.92)	0.009	0.90 (0.79–0.97)	0.023
Complications in the study	1.95 (1.38–2.87)	0.010	1.50 (1.10–2.33)	0.019
Mechanical ventilation	1.17 (0.90–1.49)	0.19	–	
H-H score (per grade)	2.22 (1.60–2.77)	< 0.001	1.85 (1.32–2.48)	< 0.001
Aneurysm size > 10 mm	2.48 (1.55–3.79)	0.012	2.03 (1.22–3.72)	0.041
ICH	1.36 (0.80–2.15)	0.28	–	
IVH sum score (per unit increase)	1.75 (1.22–3.02)	0.015	1.48 (1.05–2.93)	0.040
APACHE-II score (per unit increase)	1.18 (1.09–1.30)	0.009	1.06 (1.01–1.21)	0.039
Hydrocephalus	2.05 (1.48–3.02)	0.003	1.55 (1.21–2.43)	0.011
TCD cerebral vasospasm	1.78 (1.18–2.56)	0.009	1.42 (1.04–2.02)	0.021
DCI	2.21 (1.43–3.08)	< 0.001	1.76 (1.29–2.55)	0.005
Glucose (per unit increase)	1.07 (1.01–1.18)	0.042	1.04 (0.99–1.21)	0.093
CRP (per unit increase)	1.04 (1.01–1.07)	0.011	1.03 (1.01–1.08)	0.032
FABP 4(per unit increase)	1.11 (1.08–1.14)	< 0.001	1.07 (1.02–1.13)	0.001

*OR* odds ratio, *CI* confidence interval, *CRP* C-reactive protein, *APACHE-II* 5 Acute Physiology and Chronic Health Evaluation II, *IVH* intraventricular hemorrhage, *DCI* delayed cerebral ischemia, *ICH* intracerebral hemorrhage, *CRP* C-reactive protein, *TCD* transcranial Doppler, *H-H* Hunt–Hess

^a^Multivariable model included significant risk factors which are confirmed in the univariate analysis

**Fig. 4:**
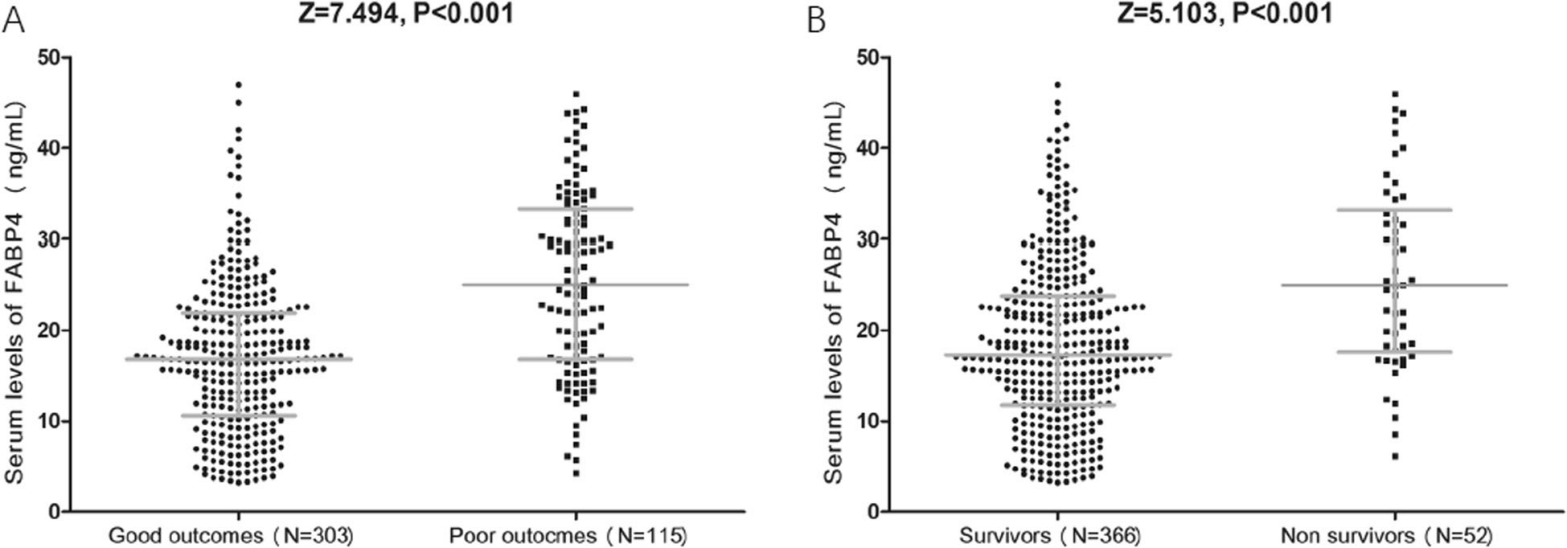
Serum levels of FABP4 in different groups in the follow-up. **a** Serum levels of FABP4 in SAH patients with poor outcome and good outcome. **b** FABP4 levels in survivors and non-survivors of SAH. Outcome was obtained using the Glasgow Outcome Scale (GOS) score ranging from 1 to 5. The outcome was dichotomized as poor with a GOS score of 1–3 points and good with a GOS score of 4–5 points. SAH = subarachnoid hemorrhage; FABP4 = fatty acid–binding protein 4

The serum levels of FABP4 in SAH patients with poor outcomes were significantly higher than in patients with good outcomes (25.0 ng/ml [IQR 16.8–33.3] vs. 16.8 ng/ml [IQR 10.6–21.9], respectively; Fig. 4a). Elevated FABP4 was also associated with increased risk of poor outcome (Table 2). The poor outcome increased significantly across increasing FABP4 quartiles, from 9.3 to 51.9% (Table 3). The 3rd and 4th quartiles of FABP4 were also compared against Q1, and elevated FABP4 status (≥ 24.9 ng/ml) was found to be related to poor outcome (Table 3).

**Table 3:** Multivariate logistic regression analysis for poor outcomes according to FABP4 quartiles

FABP4^a^	Poor/*N* (%)	Crude OR (95% CI), *P*^#^	Multivariable-adjusted^b^, *P*^#^
Quartile 1	10/107 (9.3)	Reference	Reference
Quartile 2	23/105 (21.9)	2.72 (1.22–6.05), 0.012	1.92 (0.93–5.93), 0.072
Quartile 3	28/102 (27.5)	3.67 (1.68–8.03), 0.001	2.75 (1.55–6.03), 0.021
Quartile 4	54/104 (51.9)	10.48 (4.92–22.31), < 0.001	5.68 (2.83–10.02), < 0.001
Elevated vs. normal	82/206 vs. *33*/212	3.79 (2.38–6.02), < 0.001	2.89 (1.60–6.15), 0.009

*OR* odds ratio, *CI* confidence interval, *CRP* C-reactive protein, *APACHE-II* 5 Acute Physiology and Chronic Health Evaluation II, *IVH* intraventricular hemorrhage, *DCI* delayed cerebral ischemia, *ICH* intracerebral hemorrhage, *CRP* C-reactive protein, *TCD* transcranial Doppler, *H-H* Hunt–Hess

^a^FABP4 in quartile 1 (< 12.5 ng/ml), quartile 2 (12.5–18.2 ng/ml), quartile 3 (18.3–24.9 ng/ml), and quartile 4 (> 24.9 ng/ml). Elevated FABP4 level was defined as ≥ 24.9 ng/ml (3rd quartile)

^b^Adjusted for those significant risk factors which confirmed in the univariate analysis (Table 2), including age, hypertension, cardiovascular comorbidities, a history of nicotine abuse, surgical clip, coiling, complications in the study, H-H score, aneurysm size > 10 mm, APACHE-II score, IVH sum score, hydrocephalus, cerebral vasospasm, DCI, glucose, and CRP

^#^*P* value for the trend < 0.001

The cutoff value of FABP4 to predict poor outcome was 22.0 ng/ml, which provided the highest sensitivity (62.6%) and specificity (71.5%). With an AUC of 0.74 (95% CI 0.68–0.79), FABP4 had a greater ability to predict poor outcome compared with CRP (AUC 0.65, 95% CI 0.59–0.72; *P* < 0.001), glucose (AUC 0.58, 95% CI 0.51–0.65; *P* < 0.001), age (AUC 0.68, 95% CI 0.61–0.75; *P* = 0.001), and H-H grade (AUC 0.70, 95% CI 0.63–0.76; *P* = 0.021). There was a significant difference in the AUC between conventional risk factors and FABP4 plus conventional risk factors (difference 0.024, 95% CI 0.018–0.032; Table 4). Further, the inclusion of FABP4 in the prediction model of established risk factors for the prediction of poor outcome enhanced the NRI (*P* = 0.003) and integrated discrimination improvement index (*P* = 0.02), confirming the effective reclassification and discrimination (Table 4).

**Table 4:** Serum concentrations of FABP4 at admission prediction of poor outcomes and mortality with AUROC

End points	AUROC					
	FABP4	Risk factors ^a^	Risk factors with FABP4 ^a^	Incremental area (*P*)^b^	NRI (*P*)	IDI (*P*)
Poor outcomes	0.737	0.794	0.818	0.024 (0.01)	0.201 (0.003)	0.022 (0.02)
Mortality	0.709	0.779	0.794	0.015 (0.03)	0.178 (0.009)	0.018 (0.08)

*IDI* integrated discrimination improvement, *NRI* net reclassification improvement, *CI* confidence interval, *APACHE-II* 5 Acute Physiology and Chronic Health Evaluation II, *IVH* intraventricular hemorrhage, *DCI* delayed cerebral ischemia, *CRP* C-reactive protein, *TCD* transcranial Doppler, *H-H* Hunt–Hess

^a^Included those significant risk factors which confirmed in the multivariate analysis (Table 2), including age, surgical clip, coiling, complications in the study, H-H score, aneurysm size > 10 mm, APACHE-II score, IVH sum score, hydrocephalus, cerebral vasospasm, DCI, and CRP

^b^Comparison of AUROCs: established risk factors without FABP4 levels vs. established risk factors with FABP4 levels

### Association of serum levels of FABP4 with mortality

The serum FABP4 levels in 52 patients who died were higher than in surviving patients (24.9 ng/ml [IQR 18.2–32.7] vs. 17.2 ng/ml [IQR 11.7–23.7]; Fig. 4b). Elevated FABP4 was associated with increased risk of mortality, and the risk increased by 8% (OR 1.08, 95% CI 1.05–1.12; *P* < 0.001) and 5% (OR 1.05, 95% CI 1.01–1.12; *P* = 0.003) in univariate model and multivariable analysis, respectively, for each 1 unit (ng/ml) increase in serum concentration. The 3rd and 4th quartiles of FABP4 were compared against Q1, which showed that elevated FABP4 status (≥ 24.9 ng/ml) was linked to mortality (Table 5).

**Table 5:** Multivariate logistic regression analysis for mortality according to FABP4 quartiles

FABP4^a^	Death/*N* (%)	Crude OR (95% CI), *P*^#^	Multivariable-adjusted^b^, *P*^#^
Quartile 1	4/107 (3.7)	Reference	Reference
Quartile 2	10/105 (9.5)	2.71 (0.82–8.93), 0.090	–
Quartile 3	13/102 (12.7)	3.76 (1.18–11.95), 0.017	2.59 (1.42–6.11), 0.042
Quartile 4	25/104 (24.0)	8.15 (2.73–24.37), < 0.001	4.55 (2.54–9.13), < 0.001
Elevated vs. normal	38/206 vs. *14*/212	3.20 (1.68–6.11), < 0.001	2.26 (1.30–5.77), 0.011

^a^FABP4 in quartile 1 (< 12.5 ng/ml), quartile 2 (12.5–18.2 ng/ml), quartile 3 (18.3–24.9 ng/ml), and quartile 4 (> 24.9 ng/ml). Elevated FABP4 level was defined as ≥ 24.9 ng/ml (3rd quartile)

^b^Adjusted for those significant risk factors which confirmed in the univariate analysis (Table 2), including age, hypertension, cardiovascular comorbidities, a history of nicotine abuse, surgical clip, coiling, complications in the study, H-H score, aneurysm size > 10 mm, APACHE-II score, IVH sum score, hydrocephalus, cerebral vasospasm, DCI, glucose, and CRP

^#^*P* value for the trend < 0.001

*OR* odds ratio, *CI* confidence interval, *CRP* C-reactive protein, *APACHE-II* 5 Acute Physiology and Chronic Health Evaluation II, *IVH* intraventricular hemorrhage, *DCI* delayed cerebral ischemia, *ICH* intracerebral hemorrhage, *CRP* C-reactive protein, *TCD* transcranial Doppler, *H-H* Hunt–Hess

The cutoff value of FABP4 to predict mortality was 24.5 ng/ml, which provided the highest sensitivity (54.0%) and specificity (77.5%). FABP4 showed a greater ability to predict mortality (AUC 0.71, 95% CI 0.6 to − 0.78) compared with CRP (AUC 0.66, 95% CI 0.61–0.73; *P* = 0.001), glucose (AUC 0.60, 95% CI 0.54–0.66; *P* < 0.001), age (AUC 0.64, 95% CI 0.59–0.72; *P* < 0.001), and the range of H–H grade (AUC 0.73, 95% CI 0.66–0.80; *P* = 0.11). There was a significant difference in the AUC between conventional risk factors and FABP4 plus conventional risk factors (difference 0.015, 95% CI 0.011–0.020; Table 4). The inclusion of FABP4 in the prediction model of established risk factors for the prediction of mortality increased the NRI (*P* = 0.009), confirming the effective reclassification and discrimination (Table 4).

**Fig. 5:**
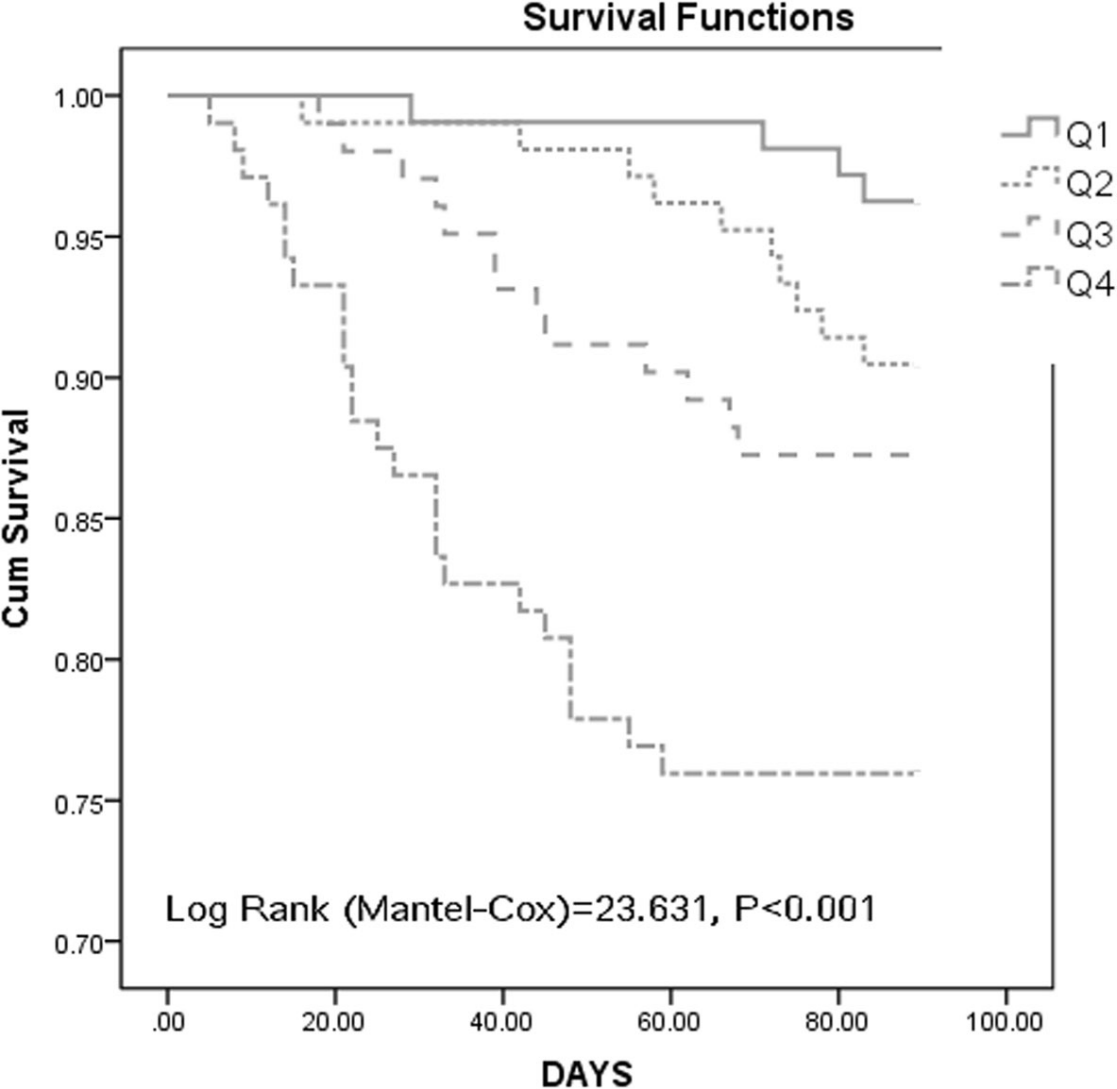
Kaplan–Meier survival curves for FABP 4 quartiles. FABP4 in quartile 1 (< 12.5 ng/ml), quartile 2 (12.5–18.2 ng/ml), quartile 3 (18.3–24.9 ng/ml), and quartile 4 (> 24.9 ng/ml). FABP4 = fatty acid–binding protein 4

Kaplan–Meier analysis according to FABP4 quartiles showed that patients with the highest quartile levels of FABP4 had a higher risk of death, in contrast to patients with FABP4 levels in the 1st, 2nd, and 3rd quartiles (log-rank test, *P* < 0.001; Fig. 5).

### Sub-group analysis

The subgroup of patients with hypertension showed higher FABP4 levels compared with patients with no hypertension (18.6 ng/ml [IQR 13.3–26.5] vs. 16.8 ng/ml [IQR 11.4–22.6]; *P* < 0.001). Similarly, the subgroup of patients with cardiovascular comorbidities showed higher FABP4 levels compared with patients with no cardiovascular comorbidities (19.2 ng/ml [IQR 13.5–27.3] vs. 16.5 ng/ml [IQR 11.1–22.2]; *P* < 0.001). At follow-up, 103 patients were diagnosed with hydrocephalus. The serum levels of FABP4 in those cases were higher than in other patients (21.5 ng/ml [IQR 15.1–28.2] vs. 17.2 ng/ml [11.4–23.2], respectively; *P* < 0.001). FABP4 was linked to hydrocephalus, and the unadjusted and adjusted risks were increased by 7% (OR 1.07, 95% CI 1.04–1.10) and 5% (OR 1.05, 95% CI 1.00–1.10), respectively.

TCD cerebral vasospasm was also observed in 92 patients. The serum level of FABP4 in patients with TCD cerebral vasospasm was higher than in other patients (22.5 ng/ml [IQR 15.9–30.2] vs. 16.8 ng/ml [IQR 10.7–22.8]; *P* < 0.001). FABP4 was also related to cerebral vasospasm, and the unadjusted and adjusted risks were increased by 8% (OR 1.08, 95% CI 1.04–1.15) and 5% (OR 1.05, 95% CI 1.00–1.12), respectively. FABP4 was associated with DCI, and the unadjusted and adjusted risks were increased by 10% (OR 1.10, 95% CI 1.03–1.18) and 6% (OR 1.06, 95%CI 1.01–1.11), respectively. The serum FABP4 levels in aSAH with DCI were higher than in those patients without DCI (23.3 ng/ml [IQR 15.9–30.2] vs. 16.5 ng/ml [IQR 10.1–21.4]; *P* < 0.001).

## Discussion

FABP4 is an adipokine recently shown to be linked with cardiovascular and metabolic diseases [11]. aSAH is a serious medical condition in which outcome can be dramatically impacted by early, aggressive, expert care [23]. In the present study, we first evaluated serum levels of FABP4 in Chinese patients with aSAH to investigate the relationship between poor outcome/mortality and FABP4 levels. We found that higher FABP4 levels were correlated with an increased risk of poor prognosis in patients with aSAH. This correlation remained significant after adjusting for established risk factors. In addition, FABP4 showed promise as a biomarker for secondary neurological insults, including hydrocephalus, TCD cerebral vasospasm, and DCI, after SAH.

The prognostic value of FABP4 in cardiovascular and cerebrovascular diseases was previously reported. Tu et al. [24] showed that FABP4 was a novel independent prognostic marker in patients with ischemic stroke. FABP4 was also related to atherogenesis and poor outcome in acute ischemic stroke patients with carotid atherosclerosis [25]. Further, circulating FABP4 was used as a prognostic biomarker in patients with acute coronary syndrome [26] and stable peripheral artery disease (PAD) [27]. Similarly, a long-term prognostic role of FABP4 in patients with coronary heart disease was proposed [28], while circulating FABP4 levels were reported to be an independent biomarker of various mortality in type 2 diabetes [29]. Interestingly, other types of FABP have also been examined in SAH patients. For example, Zanier et al. [30] reported that heat-FABP levels were associated with brain injury after SAH and could help predict long-term prognosis. Further, heat-FABP was used as a biomarker to identify the risk of poor outcome in patients with aSAH [31].

It remains unclear how FABP4 plays a role in the pathogenesis of aSAH. We found that FABP4 was associated with admission H-H scores, which are related to a poor prognosis. Further, secondary neurological insults, such as vasospasm and DCI, after SAH may cause poor outcomes [32]. Rodríguez-Rodríguez et al. [33] reported that neurological status on admission, the magnitude of the initial bleeding, and cerebral vasospasm were directly linked to poor prognosis in SAH patients. Further, a study of Chinese patients with aSAH showed that DCI was related to severe clinical course and poor prognosis at both short- and long-term follow-up [34]. In the present study, FABP4 levels were also correlated with increased risk of hydrocephalus, TCD cerebral vasospasm, and DCI after SAH. Speculatively, FABP4 may cause adverse prognosis in SAH patients by affecting admission severity scores and secondary neurological insults. Nevertheless, the correlation of FABP4 with aSAH remained after adjusting for those factors in the regression analysis.

Alternatively, activation of the inflammatory generating complement system may play a pathogenic role in SAH. A role for FABP4 in macrophage cholesterol trafficking and associated inflammation was proposed [25, 35]. Circulating FABP4 levels are also related to vascular inflammation [36]. Further, postoperative CRP was a useful prognostic biomarker for both poor outcome and vasospasm in aSAH patients [37], while Chen et al. [38] found that macrophage migration inhibitory factor provided information on inflammation, brain injury, and prognosis after aSAH. Spontaneous elevations in mean arterial blood pressure are also correlated with poorer outcomes in aSAH [39]. Further, FABP4 can increase blood pressure and atherogenic metabolic phenotype in hypertensive patients [40], while Zoerle et al. [41] reported that intracranial pressure was linked to early brain injury and mortality. Interestingly, experimental studies have shown that FABP4 deficiency can protect mice against ischemia/reperfusion-induced cardiac injury through activation of the endothelial nitric oxide synthases–nitric oxide pathway, and reduction of superoxide anion production [42]. Further, the anti-inflammatory phenotype of FABP4 null mice increased the expression of uncoupling protein 2 and SIRT3 through increased intracellular mono-unsaturated fatty acids [43]. Finally, higher levels of FABP4 can lead to cell death, with elevated expression of FABP4 promoting saturated fatty acid–induced macrophage cell death through increasing ceramide production [44].

### Strengths and limitations

The main study strength of this study was the inclusion of multiple confounding factors in a multivariate analysis, which revealed the relationship between FABP4 and SAH outcomes. In addition, we used a variety of statistical analyses to make our results more representative and reliable. However, there are some potential limitations. First, a causal relationship between FABP4 and poor prognosis could not be confirmed because of the cross-sectional research design. Further studies are required to determine whether normalizing serum FABP4 levels can improve prognosis. Interestingly, Furuhashi et al. [45] confirmed that a reduction of FABP4 levels by angiotensin II receptor blockers was related to suppression of cardiovascular events. However, we were unable to obtain longitudinal data of biomarkers, as the serum levels of FABP4 were only tested once after disease onset, without follow-up to detect FABP4 levels at different disease stages (e.g., acute stage or remission stage). Thus, we could not determine whether SAH caused increased serum excretion of FABP4 or whether increased FABP4 levels existed prior to SAH. Third, it would be interesting to compare non-ruptured aneurysm patients with matched healthy controls to address the problem of measuring FABP4 levels after aneurysm rupture on admission. Fourth, because of technical limitations, we did not test other adipokine biomarkers such as adiponectin and leptin. Fifth, the actual cause of the high level of FABP4 in aSAH remains unclear. Finally, we only investigated the prognosis of the patients at 3 months after admission, and future long-term follow-up studies are required.

## Conclusions

Elevated serum levels of FABP4 were related to poor outcome and mortality in a cohort of patients with aSAH. FABP4 may be a useful predictor of aSAH, independent of established conventional risk factors.

## Data Availability

Please contact corresponding author for data requests.

## Abbreviations

APACHE: Acute Physiology and Chronic Health Evaluation
aSAH: Aneurysmal SAH
AUC: Area under the curve
BMI: Body mass index
CI: Confidence interval
CT: Computed tomography
DCI: Delayed cerebral ischemia
ELISA: Enzyme-linked immunosorbent assay
FABP4: Fatty acid–binding protein 4
GOS: Glasgow Outcome Scale
H-H: Hunt–Hess
IDI: Integrated discrimination improvement
IQR: Interquartile ranges
LVH: Left ventricular hypertrophy
NRI: Net reclassification improvement
OR: Odds ratios
PAD: Peripheral artery disease
ROC: Receiver operating characteristic
SAH: Subarachnoid hemorrhage
TCD: Transcranial Doppler
